# Characterization of the Polyetheretherketone Weldment Fabricated via Rotary Friction Welding

**DOI:** 10.3390/polym15234552

**Published:** 2023-11-27

**Authors:** Chil-Chyuan Kuo, Hua-Xhin Liang, Song-Hua Huang

**Affiliations:** 1 Department of Mechanical Engineering, Ming Chi University of Technology, No. 84, Gungjuan Road, New Taipei City 24301, Taiwan; 2 Research Center for Intelligent Medical Devices, Ming Chi University of Technology, No. 84, Gungjuan Road, New Taipei City 24301, Taiwan; 3 Department of Mechanical Engineering, Chang Gung University, No. 259, Wenhua 1st Rd., Guishan District, Taoyuan City 33302, Taiwan; 4 Center of Reliability Engineering, Ming Chi University of Technology, No. 84, Gungjuan Road, Taishan District, New Taipei City 24301, Taiwan; 5 Li-Yin Technology Co., Ltd., No. 37, Lane 151, Section 1, Zhongxing Road, Wugu District, New Taipei City 24301, Taiwan

**Keywords:** polyether ether ketone, rotational friction welding, burn-off length, bending strength, cycle time

## Abstract

Polyether ether ketone (PEEK) is frequently employed in biomedical engineering due to its biocompatibility. Traditionally, PEEK manufacturing methods involve injection molding, compression molding, additive manufacturing, or incremental sheet forming. Few studies have focused on rotational friction welding (RFW) with PEEK plastics. Based on years of RFW practical experience, the mechanical properties of the weldment are related to the burn-off length. However, few studies have focused on this issue. Therefore, the main objective of this study is to assess the effects of burn-off length on the mechanical properties of the welded parts using PEEK polymer rods. The welding pressure can be determined by the rotational speed according to the proposed prediction equation. The burn-off length of 1.6 mm seems to be an optimal burn-off length for RFW. For the rotational speed of 1000 rpm, the average bending strength of the welded parts was increased from 108 MPa to 160 Mpa, when the burn-off length was increased from 1 mm to 1.6 mm and the cycle time of RFW was reduced from 80 s to 76 s. A saving in the cycle time of RFW of about 5% can be obtained. The bending strength of the welded part using laser welding is lower than that using RFW, because only the peripheral material of the PEEK cylinder was melted by the laser.

## 1. Introduction

Polyether ether ketone (PEEK) has exceptional properties, such as high wear, chemical, mechanical, thermal, and radiation resistance. Thus, PEEK is widely employed in various applications, including pumps, bearings, or piston parts. PEEK is also used in medical implants. Therefore, PEEK has been frequently employed for human bone replacement due to its superior biomechanical properties [1], which have potential applications in the biomedical and aerospace industries [2,3]. PEEK exhibits outstanding thermal resilience and mechanical characteristics, showcasing its high-performance crystalline polymer nature.

Khoran et al. [4] evaluated the effects of reinforcing the fiber type on the grinding process of PEEK’s composites. It was found that the tangential and normal grinding forces increased up to 67% using severe cutting conditions. Lee et al. [5] investigated the influence of jet pressure on the bond strength of PEEK. Results showed that adhesive failure is the primary mode for all groups and provides sufficient bonding durability between dental resin cement and PEEK. As a result, PEEK seems to be a promising alternative to metal in dentistry [6]. Vaddamanu et al. [7] investigated the fitting surface deformation of PEEK clasps and compared them with cobalt–chromium clasps. Results showed that the deformation of PEEK clasps’ fitting surface is lower than for cobalt–chromium clasps. This finding implies that opting for PEEK as a clasp in the removable partial denture framework is a viable choice for aesthetic reasons. Bontempi et al. [8] measured the nanomechanics of medical-grade PEEK using atomic force microscopy. Results showed that two localized distributions of moduli at about 0.6 and 0.9 GPa were found. This noteworthy discovery offers insights into the ideal design of nanostructures, paving the way for advanced biomedical applications. Yang et al. [9] employed molecular dynamics to simulate local vibrational features and evaluate the local crystallinity of PEEK. The observation of the 135 cm^−1^ signal was identified, showcasing its susceptibility to intermolecular interactions. Naghavi et al. [10] proposed low-stiffness PEEK hip prostheses for minimizing the stress difference after a hip replacement. Results showed that the stiffness of the PEEK implant was about 0.276 kN/mm, showing that PEEK seems to be a promising material for implants. Zhou et al. [11] investigated the shape-memory properties and actuation performances of high-temperature PEEK. Results showed that the results obtained in this study provided a design for a PEEK actuator. Baltag et al. [12] proposed a new method for the sulfonation of PEEK with a temperature of 60 °C under ultrasonication. It was found that a maximum removal efficiency of about 99% was obtained. Saravi et al. [13] studied the fatigue behavior of PEEK implant abutments. It was found that the PEEK implant abutments seem to be feasible, compared to zirconia implant abutments. Zhang et al. [14] used the single-pulsed laser to drill PEEK. Results showed that the ejection is the primary material-removal mechanism during the slow increment stage, showing the dynamic process of keyhole evolution in the drilling of PEEK using the single-pulsed laser. Bialas et al. [15] used picosecond laser treatment to obtain the surface-infused nanogold particles with PEEK. Results showed that an increase in quantity was obtained using a laser power of 15 mW. Pedroso et al. [16] investigated the tribological performance of waterborne tribological coatings based on three binders of PEEK and polyetherketone. Results suggested that PEKK and polyetherketone seem to be attractive alternatives to PEEK.

Rotary friction welding (RFW) [17] offers various benefits, such as superior joint strength and the capability to unite dissimilar materials. Traditionally, common PEEK manufacturing methods encompass additive manufacturing [18], injection molding [19,20,21,22], milling, grinding, pulse laser drilling, or incremental sheet forming, among others [23]. It is well-known that the ultrasonic welding (UW) can be used to join PEEK plastics [24,25,26]. However, the distinct drawback of this approach is that it is limited to smaller and thinner PEEK components. Based on RFW practical experience, the weldment quality was affected by the burn-off length [27]. However, very few studies have focused on this issue. Based on the above two research motivations, this study aims to characterize the polyetheretherketone weldment fabricated using RFW and evaluate the impact of the burn-off length on the weld quality of PEEK polymer rods. A non-contact thermal camera was employed to investigate the temperature changes in the weld interface during RFW. Subsequent to RFW, the mechanical properties of the welded parts were examined using shore A surface hardness and three-point bending tests. Thermal properties were also investigated using differential scanning calorimetry (DSC). The culmination of this study involves proposing a technical database on RFW, explicitly focusing on the influence of the burn-off length on weld properties.

## 2. Materials and Methods

**Figure 1:**
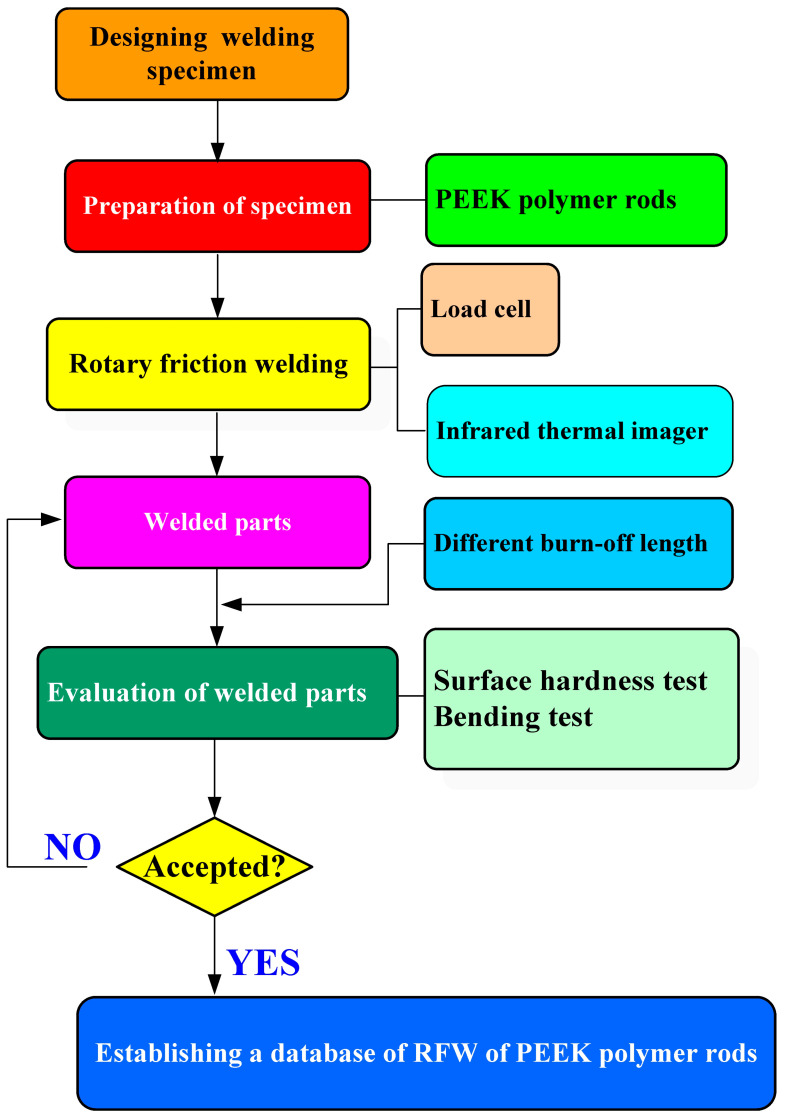
Research flowchart in this study.

Figure 1 shows the research flowchart in this study. Five rotational speeds were used in this study. In this study, a PEEK polymer rod was selected as the welding specimen. The length and diameter were about 40 mm and 20 mm, respectively. It is noteworthy that the PEEK polymer rod has a melting point of around 343 °C and a decomposition temperature of approximately 575 °C [28]. The speed of the spindle was kept at constant speeds of 1000, 1350, 2000, 3000, and 4000 rpm, respectively. The cycle time of RFW under a burn-off length of 1 mm was 90 s, which involved a friction time of 30 s, a welding time of 30 s, and a cooling time under the pressure of 30 s. The axial load and feed rate are 24.1 N and 6 mm/min, respectively. The welding pressure of RFW is 0.077 MPa. To study the effects of the burn-off length on the weldment quality of PEEK polymer rods, eight different burn-off lengths of welded parts were employed in this study, i.e., 1, 1.2, 1.3, 1.4, 1.5, 1.6, 1.8, and 2 mm.

**Figure 2:**
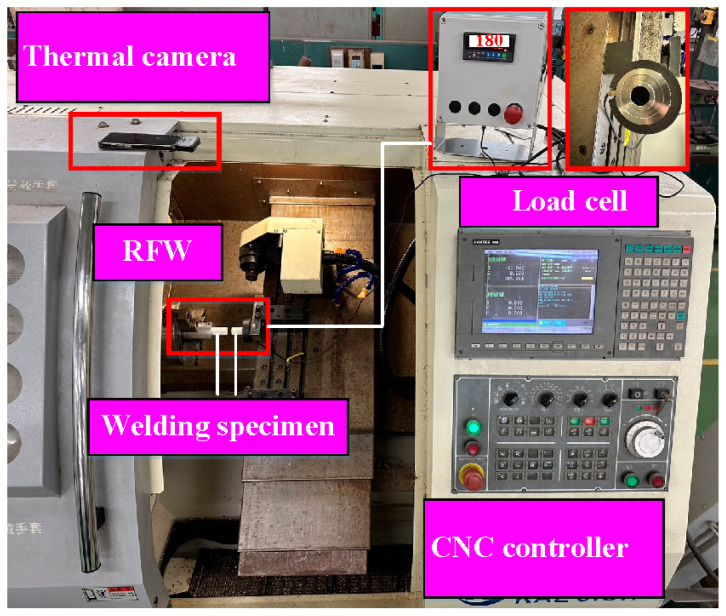
Computer numerical control lathe used for RFW.

**Figure 3:**
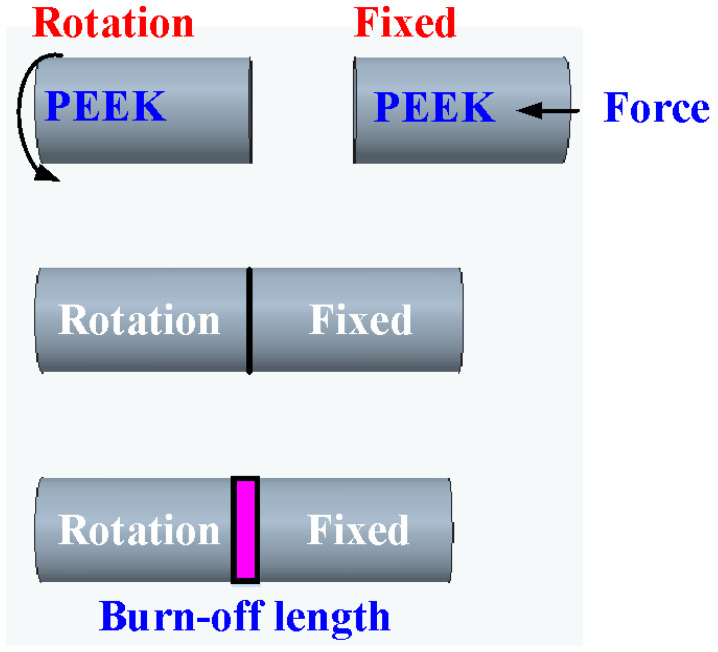
Schematic illustration of burn-off length during RFW.

**Figure 4:**
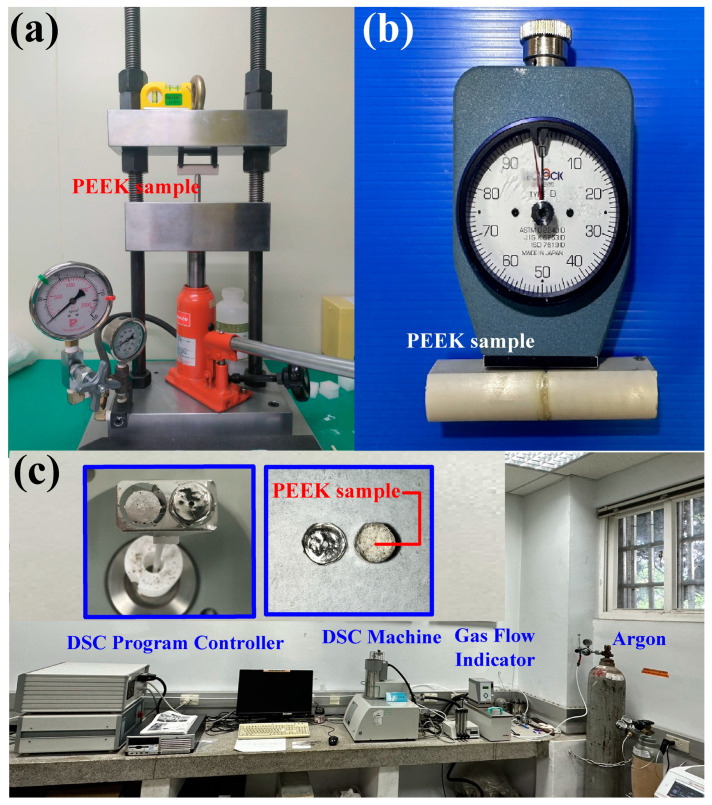
Experimental setup for (**a**) bending test, (**b**)surface hardness, and (**c**) DSC test of welded parts.

In this study, a computer numerical control (CNC) turning machine (K-45L, Kae Jiuh, Co., Inc., New Taipei City, Taiwan) was employed to perform RFW of PEEK polymer rods, as shown in Figure 2. A non-contact thermal camera (BI-TM-F01P, Panrico trading Inc., New Taipei City, Taiwan) was used to investigate the temperature changes at the weld interface during the RFW process. A load cell (ARI742, Zhiheng Industrial Co., Inc., New Taipei City, Taiwan) was used to measure the welding force applied for RFW. Figure 3 depicts a schematic illustration of the burn-off length during RFW. The welding specimen has a round cross-section with the diameter and length of 20 mm and 40 mm. One specimen is firmly held stationary. The other specimen is fixed with a chuck and is rotated at a constant rotational speed. The frictional heat is generated at the interface of two workpieces during RFW. Figure 4 shows the experimental setup for bending, surface hardness, and DSC tests of welded parts. A 1500 W pulsed fiber laser (COMET Inc., New Taipei City, Taiwan) with a wavelength of 1062 nm was also used to join PEEK polymer rods. The output power is about 40 W, and the repetition frequency of the laser is about 80 kHz. The laser welding speed and spot size are 2 mm/s and 3 mm, respectively. The three-point bending machine (RH-30, Shimadzu Inc., Kyoto, Japan) and The Shore A durometer (MET-HG-A, SEAT Inc. New Taipei City, Taiwan) were used to evaluate the weldment quality after RFW. The movement speed of the punch is about 1 mm/s. The bending strength (*σ*) can be calculated using the following Equation (1).

(1) $$\sigma =\frac{8PL}{\pi d^{3}}$$

where *P* is the applied load, *d* is the diameter of the PEEK polymer rod, and *L* is the length between two supports.

The scanning electron microscopy (FE-SEM) (JEC3000-FC, JEOL Inc. Tokyo, Japan) and optical microscope (OM) (Quick Vision 404, Mitutoyo Inc., Tokyo, Japan) were used to investigate the fracture surfaces after a series of bending tests. The thermal transitions of the weldments were examined using the differential scanning calorimetry (DSC) (STA 409 PC Luxx Simultaneous thermal analyzer, Netzsch-Gerätebau GmbH Inc., Selb, Germany). A mass of between 10 and 15 mg of the welded joint samples was placed in platinum crucibles for the DSC. The specimens were heated at a temperature ranging from 30 °C to 400 °C under a nitrogen gas flow rate of about 25 cc/min. Both the heating rate and cooling rate were 10 °C/min. A technical database was finally established about the characterization of PEEK weldment fabricated by RFW.

## 3. Results and Discussion

**Figure 5:**
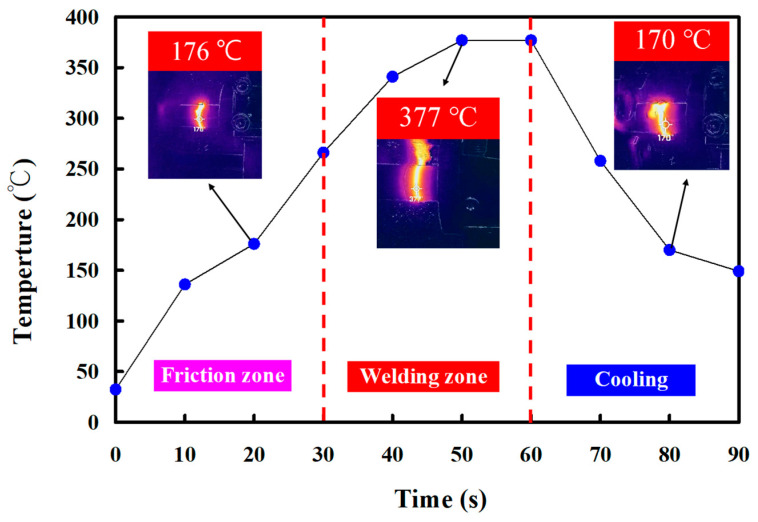
The relationship between time and weld joint temperature under the rotational speed of 1000 rpm.

**Figure 6:**
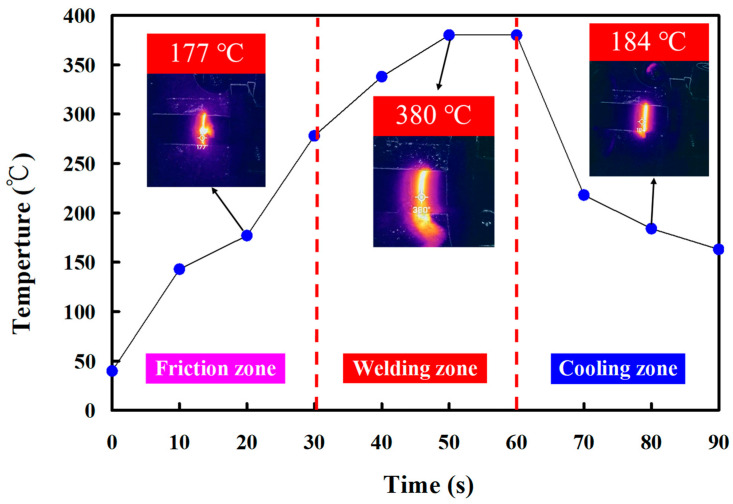
The relationship between time and weld joint temperature under the rotational speed of 1350 rpm.

**Figure 7:**
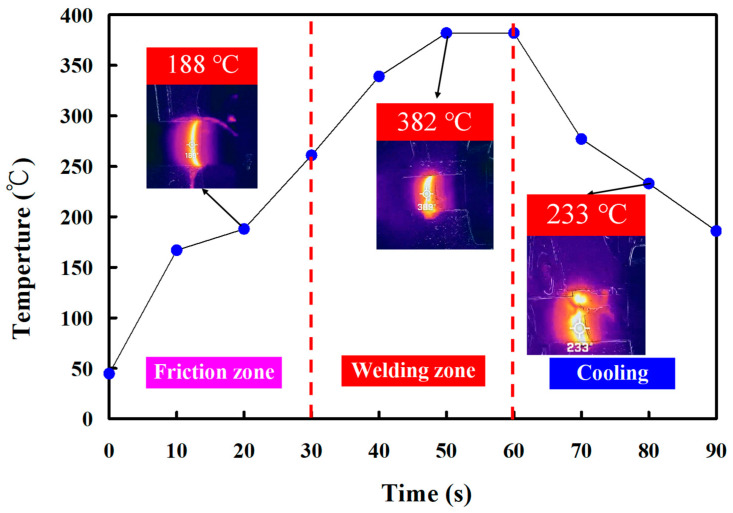
The relationship between time and weld joint temperature under the rotational speed of 2000 rpm.

**Figure 8:**
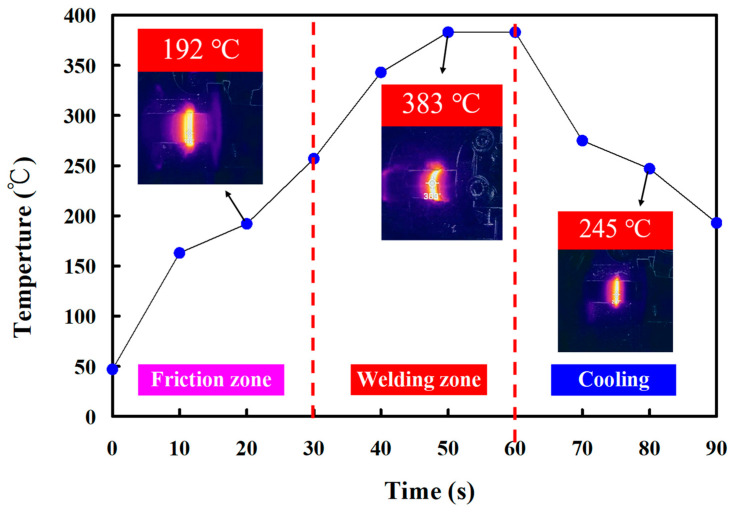
The relationship between time and weld joint temperature under the rotational speed of 3000 rpm.

**Figure 9:**
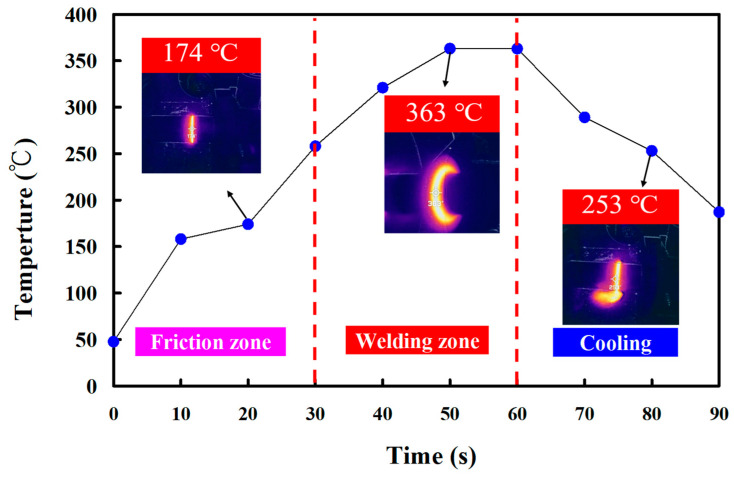
The relationship between time and weld joint temperature under the rotational speed of 4000 rpm.

In this study, five specimens were produced. Based on many years of RFW practical experience, the weldment quality was significantly affected by the maximum temperature in the weld interface during RFW of the PEEK polymer rods. This study used a thermal camera to investigate the temperature in the weld joint during RFW of PEEK polymer rods. Figure 5 shows the relationship between time and the weld joint temperature under the rotational speed of 1000 rpm. The maximum temperature in the weld interface is about 377 °C. Figure 6 shows the relationship between time and the weld joint temperature under the rotational speed of 1350 rpm. The maximum temperature in the weld interface is about 380 °C. Figure 7 shows the relationship between time and the weld joint temperature under the rotational speed of 2000 rpm. The maximum temperature in the weld interface is about 382 °C. Figure 8 shows the relationship between time and the weld joint temperature under the rotational speed of 3000 rpm. The maximum temperature in the weld interface is about 383 °C. Figure 9 shows the relationship between time and the weld joint temperature under the rotational speed of 4000 rpm. However, the peak temperature in the weld interface is only about 363 °C. The possible reason is that the friction coefficient of the material is different due to the high rotation speed. It is interesting to note that these temperatures at the weld joint were higher than the melting point of PEEK polymer materials [28].

**Figure 10:**
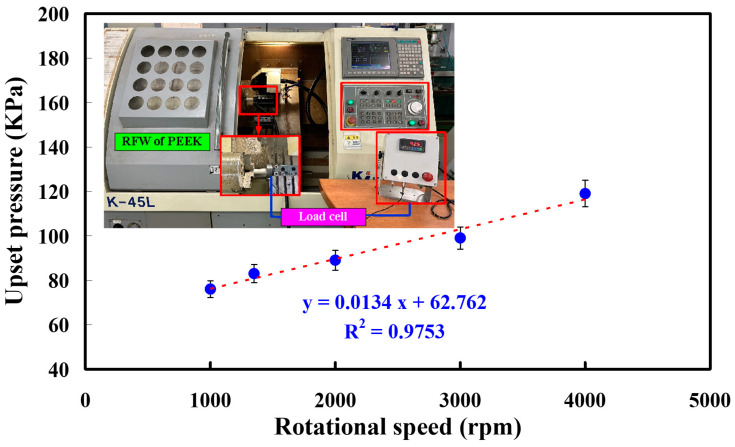
The relationship between rotational speed and welding pressure during RFW of PEEK polymer rods.

**Figure 11:**
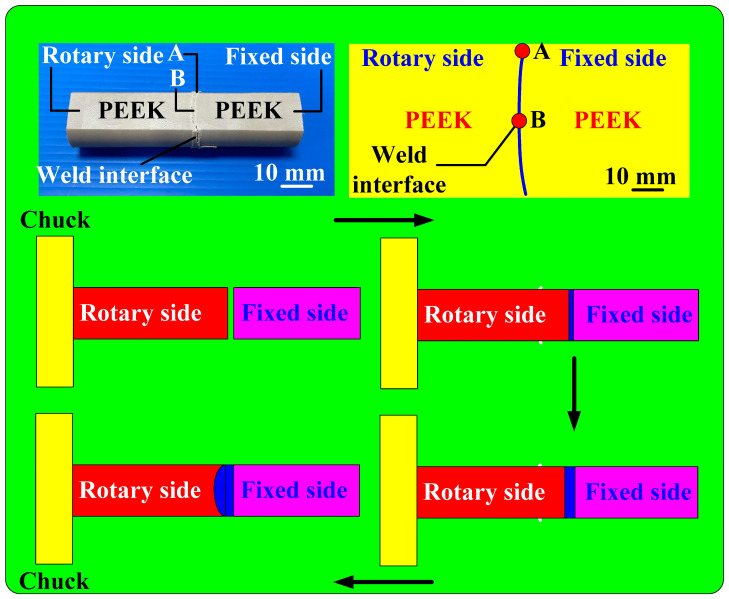
The evolution mechanism of weld interface surface after RFW of PEEK polymer rods.

Five test specimens and five rotational speeds were used to perform RFW in this study. To investigate the relationship between the rotational speed of RFW and welding force, the feed rate is fixed to perform the experiment. In this study, the feed rate is fixed at 6 mm/min. Figure 10 shows the relationship between the rotational speed and welding pressure during RFW of PEEK polymer rods. The welding force for rotational speeds of 1000, 1350, 2000, 3000, and 4000 rpm is 24.1, 26.1, 28.1, 31.4, 37.4 N, respectively. The welding pressure for rotational speeds of 1000, 1350, 2000, 3000, and 4000 rpm is 76, 83, 89, 99, and 119 kPa, respectively. For the feed rate of 6 mm/min, the welding pressure (y) can be determined by the rotational speed (x) according to the prediction equation of y = 0.0134x + 62.762 with a correlation coefficient of 0.9753. This study found that the weld interface after the RFW is a curved surface. Figure 11 shows the evolution mechanism of the weld interface surface after RFW of PEEK polymer rods, showing the change in the weld interface profile after RFW of the PEEK polymer rods. Compared to the center position B of the welding specimen, the edge position A of the welding specimen has a higher linear velocity under the same number of revolutions, which results in a higher temperature at edge position A and more material melting.

**Figure 12:**
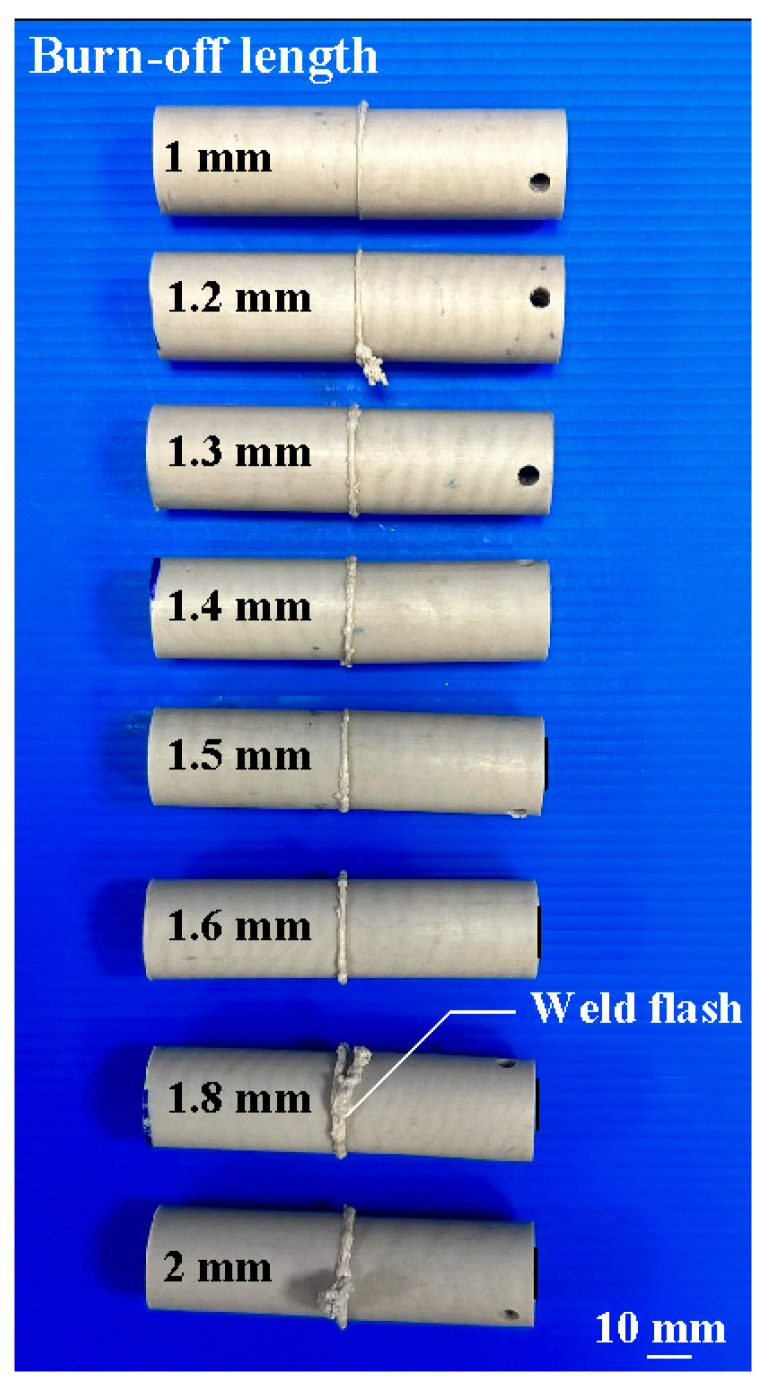
Sets of welded joints under different burn-off lengths.

**Figure 13:**
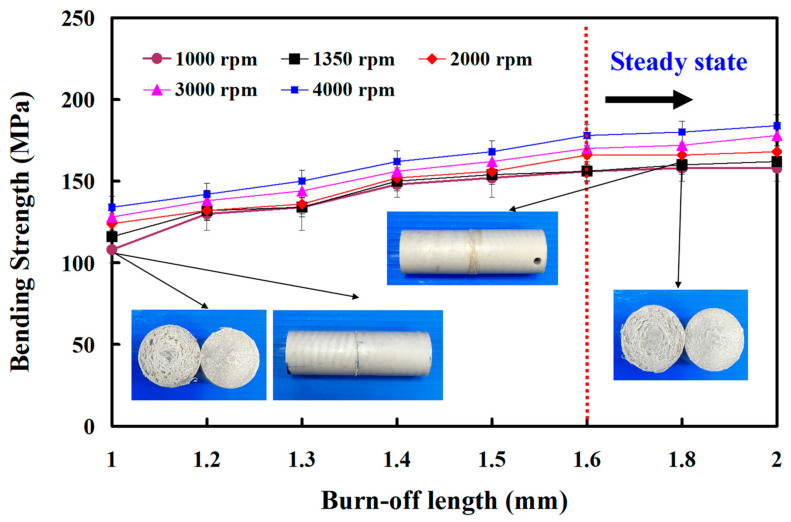
Bending strength of the welded parts after bending tests.

In this study, five specimens were performed. Figure 12 shows the sets of welded joints under different burn-off lengths. Figure 13 shows the bending strength of the welded parts after bending tests. The mean bending strength of the PEEK polymer rods is about 183 MPa. For the rotational speed of 1000 rpm, the average bending strength of the welded parts using eight different burn-off lengths for RFW is about 108 MPa, 130 MPa, 134 MPa, 148 MPa, 152 MPa, 156 MPa, 158 MPa, and 158 MPa, respectively. The cycle time of RFW is about 70 s, 72 s, 73 s, 74 s, 75 s, 76 s, 78 s, and 80 s, respectively. For the rotational speed of 1350 rpm, the average bending strength of the welded parts using eight different burn-off lengths for RFW is about 116 MPa, 132 MPa, 134 MPa, 150 MPa, 154 MPa, 156 MPa, 160 MPa, and 162 MPa, respectively. For the rotational speed of 2000 rpm, the average bending strength of the welded parts using eight different burn-off lengths for RFW is about 124 MPa, 132 MPa, 136 MPa, 152 MPa, 156 MPa, 166 MPa, 166 MPa, and 168 MPa, respectively. For the rotational speed of 3000 rpm, the average bending strength of the welded parts using eight different burn-off lengths for RFW is about 128 MPa, 138 MPa, 144 MPa, 156 MPa, 162 MPa, 170 MPa, 172 MPa, and 178 MPa, respectively. For the rotational speed of 4000 rpm, the average bending strength of the welded parts using eight different burn-off lengths for RFW is about 134 MPa, 142 MPa, 150 MPa, 162 MPa, 168 MPa, 178 MPa, 180 MPa, and 184 MPa, respectively. Based on the above results, this study found three phenomena: (a) The burn-off length of RFW has an impact on the average bending strength of the welded parts. As can be seen, the mean bending strength of the PEEK polymer rods stabilizes when the burn-off length surpasses 1.6 mm. Thus, the optimal burn-off length of 1.6 mm is suitable for cylindrical rods with a length of 40mm and a diameter of 20 mm for five rotational speeds. (b) The average bending strength of the welded parts increased from 108 MPa to 160 MPa when the burn-off length increased from 1 mm to 1.6 mm for the rotational speed of 1000 rpm. The increase in the average bending strength of the welded parts is about 48%. This result is confirmed by the thermal analyses from DSC traces.

The basis for choosing the polynomial orders is based on the correlation coefficient. A high correlation coefficient determines the polynomial orders. For the rotational speed of 1000 rpm, the bending strength of the welded parts (y) can be determined by the burn-off length (x) according to the prediction equation of y = 0.1111x^3^ − 2.8095x^2^ + 24.175x + 87.857 with a correlation coefficient of 0.9828. For the rotational speed of 1350 rpm, the bending strength of the welded parts (y) can be determined by the burn-off length (x) according to the prediction equation of y = 0.0404x^3^ − 1.4502x^2^ + 16.557x + 101.43 with a correlation coefficient of 0.975. For the rotational speed of 2000 rpm, the bending strength of the welded parts (y) can be determined by the burn-off length (x) according to the prediction equation of y = 0.0404x^3^ − 1.4502x^2^ + 16.557x + 101.43 with a correlation coefficient of 0.975. For the rotational speed of 2000 rpm, the bending strength of the welded parts (y) can be determined by the burn-off length (x) according to the prediction equation of y = −0.2121 x^3^ + 2.2446x^2^ + 1.4567x + 120.57 with a correlation coefficient of 0.9839. For the rotational speed of 3000 rpm, the bending strength of the welded parts (y) can be determined by the burn-off length (x) according to the prediction equation of y = −0.4762x^2^ + 11.476x + 116.5 with a correlation coefficient of 0.9935. For the rotational speed of 4000 rpm, the bending strength of the welded parts (y) can be determined by the burn-off length (x) according to the prediction equation of y = −0.5119x^2^ + 12.107x + 120.82 with a correlation coefficient of 0.9914.

**Figure 14:**
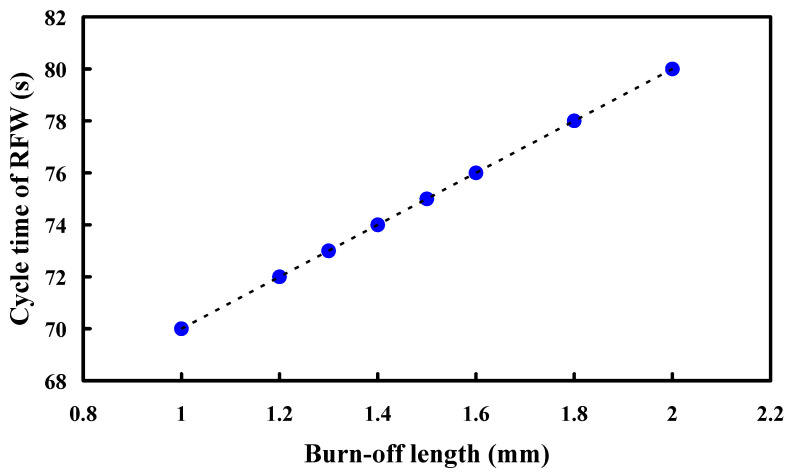
Dependence of the cycle time of RFW and burn-off length.

Figure 14 shows the dependence of the cycle time of RFW and burn-off length. It should be noted that the cycle time of RFW (y) can be determined by the burn-off length (x) according to the prediction equation of y = 10x + 60. Conventionally, the cycle time of RFW is 80 s for the burn-off length of 2 mm. According to the results obtained in this study, the burn-off length of 0.4 mm can be reduced. Thus, the cycle time of RFW can be reduced from 80 s to 76 s for five rotational speeds. The saving in the cycle time of RFW is about 5%.

**Figure 15:**
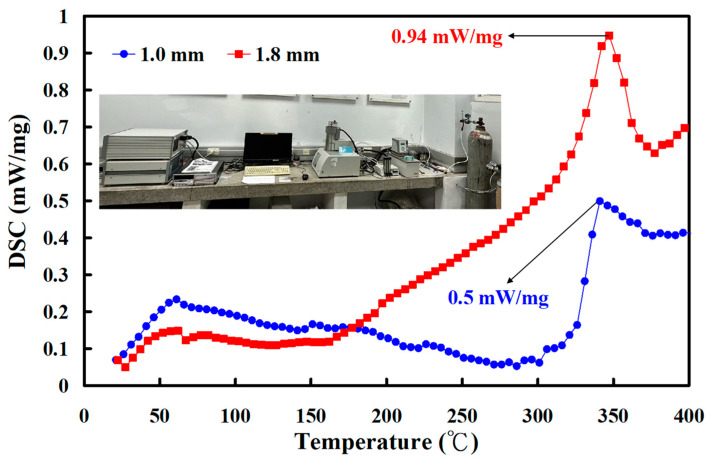
DSC traces of welded parts.

Figure 15 shows the DSC traces of welded parts. The heat capacities of welded parts fabricated using RFW with the burn-off length of 1.8 mm and 1 mm are about 0.94 mW/mg and 0.50 mW/mg, respectively [29]. This result shows the molecular orientation [30,31] in the weld interface of the welded parts fabricated using RFW with a burn-off length of 1.8 mm is higher than that of the welded parts fabricated using RFW with a burn-off length of 1 mm.

**Figure 16:**
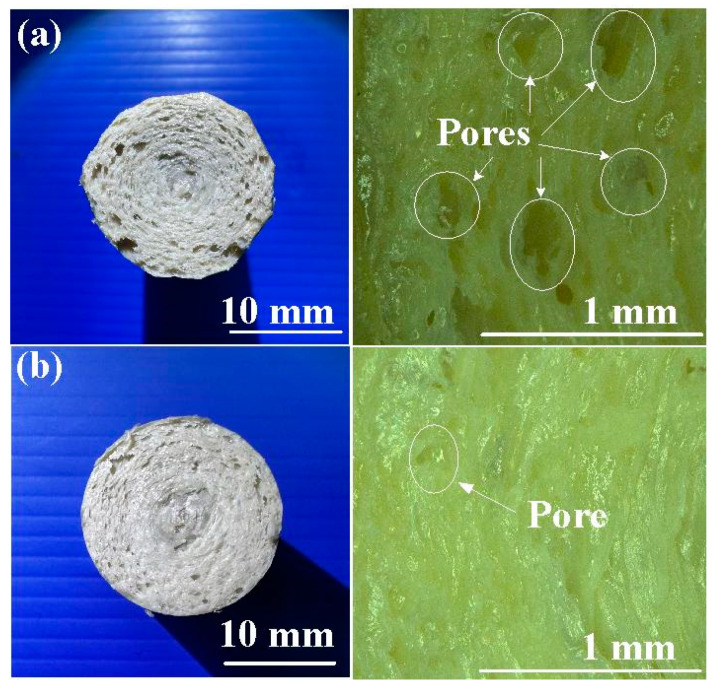
Fracture surfaces of the welded parts after bending tests with (**a**) burn-off length of 1 mm and (**b**) burn-off length of 1.6 mm.

**Figure 17:**
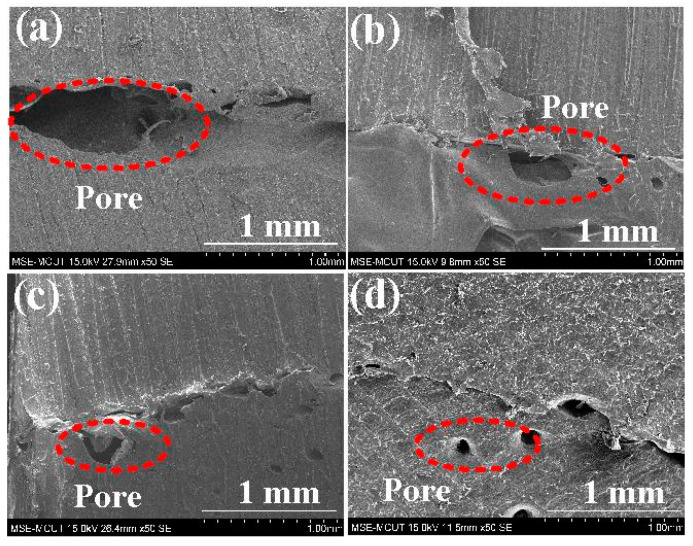
SEM micrographs of fracture surfaces of welded parts for RFW at (**a**) 1000, (**b**) 2000, (**c**) 3000, and (**d**) 4000 rpm.

Figure 16 shows the fracture surfaces of the welded parts after the bending tests. Based on the analysis of the cross-section, this study found that the number of pores in the fracture section when RFW was performed with a burn-off length of 1.6 mm was significantly less than the number of pores in the fracture section when RFW was performed using a burn-off length of 1mm. The proportion of pores in the welding area was reduced from approximately 13% to 6%. Figure 17 shows the SEM micrographs of fracture surfaces of welded parts for RFW at 1000, 2000, 3000, and 4000 rpm. It should be noted that the pores were observed in the fracture surfaces of welded parts [32]. The emergence of these pores can be attributed to the probable decomposition of materials [33]. As the rotational friction welding speed rises, the maximum temperature of the weld bead also increases. Consequently, both the fluidity and centrifugal force of the molten material experience a boost. Consequently, a noticeable reduction in pores occurs, thereby enhancing the mechanical properties of the welded components.

**Figure 18:**
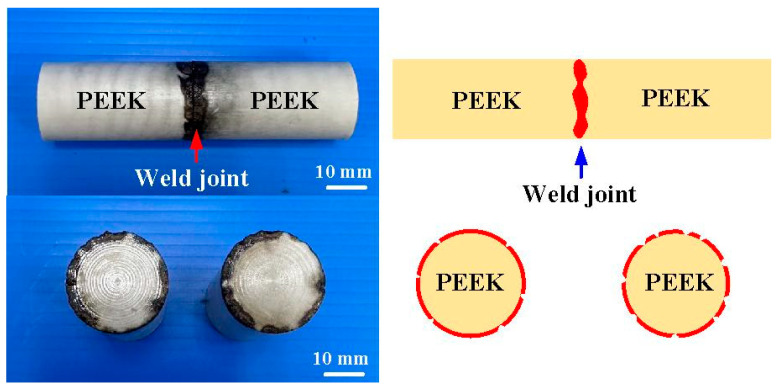
A typical welded part for laser welding of PEEK polymer rods.

**Figure 19:**
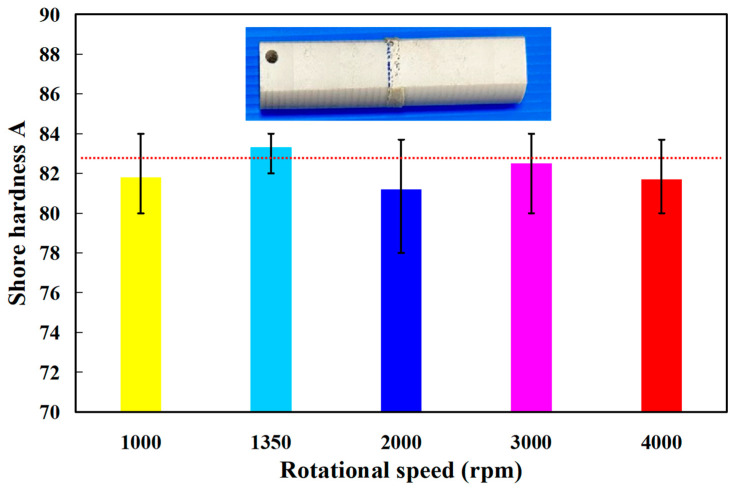
Surface hardness of the weld joint.

The distinct advantage of laser welding is its precision. To evaluate whether a laser can be used to weld PEEK polymer rods, a pulsed fiber laser with an output power of about 40 W was used to weld PEEK polymer rods. Figure 18 shows a typical welded part under laser welding of PEEK polymer rods. The results showed that only the peripheral material of the PEEK cylinder was melted by the laser. The bending strength of the welded part using laser welding is lower than that using RFW. The average bending strength is about 46 MPa. Therefore, the laser is not suitable for PEEK cylindrical welding. Figure 19 shows the surface hardness of the weld joint. There are 10 measurement points at the weld interface. As can be seen, the average Shore hardness (SH) A in the weld interface is about SH 83 for five rotational speeds. The average Shore hardness A of five welded parts is about SH 82, SH 83, SH 81, SH 83, and SH 82, respectively.

This study utilizes a CNC turning machine [34,35,36] to weld PEEK polymer rods. The advantage of this approach is that it is suitable to join large or thicker parts compared with UW [24,25,26]. In addition, the proposed method provides low environmental pollution and energy consumption compared with conventional arc welding [37]. Therefore, the proposed method has industrial applicability and complies with the sustainable development goals 7 and 12 [38,39,40]. The simulation software [41,42] is also recommended to predict the optimum process parameters of RFW, such as the rotational speed, feed rate, axial pressure, welding time, or friction time. In addition, integrating RFW with automation and robotics systems [43] is also a promising research topic because it could enhance the production efficiency and consistency [44]. These are interesting research topics and are currently being investigated.

## 4. Conclusions

Traditionally, PEEK components were predominantly produced through injection molding or compression molding. Nevertheless, there has been limited attention given to the process of RFW involving PEEK polymer rods. Based on practical RFW experience, it has been observed that the burn-off length plays a crucial role in influencing the mechanical properties of the welded components. Consequently, the main objective of this study was to examine how the burn-off length affects the mechanical properties of welded parts made from PEEK polymer rods. The primary findings derived from the experimental investigation in this study can be summarized as follows:

1. The welding pressure (y) can be determined by the rotational speed (x) according to the prediction equation of y = 0.0134x + 62.762 with a correlation coefficient of 0.9753.
2. The optimal burn-off length of 1.6 mm seems suitable for cylindrical rods with a length of 40 mm and a diameter of 20 mm for five rotational speeds.
3. For the rotational speed of 1000 rpm, the average bending strength of the welded parts increased from 108 MPa to 160 MPa when the burn-off length increased from 1 mm to 1.6 mm, and a saving in the cycle time of RFW about 5% can be obtained since the cycle time of RFW was reduced from 80 s to 76 s.
4. The peak temperature in the weld interface for a rotational speed of 4000 rpm is only about 363 °C, which was attributed to the friction coefficient of the material being different due to the high rotation speed.
5. The bending strength of the welded component through laser welding is inferior compared to that achieved using RFW. This discrepancy arises from the fact that the fiber laser only melts the peripheral material of the PEEK cylinder during the laser welding process.

## Data Availability

Data are contained within the article.
